# HbS-Sicilian (*δβ*)^0^-Thalassemia: A Rare Variant of Sickle Cell

**DOI:** 10.1155/2017/9265396

**Published:** 2017-09-17

**Authors:** Grace Onimoe, Genine Smarzo

**Affiliations:** Cleveland Clinic Lerner College of Medicine (CCLCM), Case Western University, 9500 Euclid Avenue, Cleveland, OH 44195, USA

## Abstract

Sickle cell disease (SCD) is caused by a mutation in the sixth codon of the *β*-globin gene on chromosome 11, which leads to a single amino acid substitution (glutamine to valine). Sickle-(*δβ*)^0^-thalassemia is a rare variant of sickle cell disease (delta-beta thalassemia occurring in association with sickle hemoglobin, HbS), sparsely reported in literature, and has been associated with symptomatology necessitating careful monitoring and follow-up. We describe a patient who presented with a newborn screen reported as “FS” and a negative family history for sickle cell disease and sickle cell trait. Subsequent gene sequencing studies demonstrated the presence of Sickle-(*δβ*)^0^-thalassemia. Clinical course has remained relatively stable for this patient now at 18 months of age without any SCD related symptomatology or complications. As this is a rare variant of SCD with potential complications, it is important to establish diagnosis towards planning comprehensive care.

## 1. Introduction

SCD is a multisystem disease, associated with episodes of acute illness and progressive organ damage and is one of the most common severe monogenic disorders worldwide [1, 2].

Sickle cell anemia (HbSS/HbS-Beta thalassemia zero) accounts for 70% of cases of sickle cell disease in populations of African ethnicity, with most of the remainder having hemoglobin SC disease (HBSC disease) due to the coinheritance of the *β*^s^ and *β*^c^ alleles [2, 3]. HbS/*β*-thalassemia occurs when *β*^s^ is inherited with a *β*-thalassemia allele and is a variable disorder depending on the type of *β*-thalassemia mutation [2]. Ten further genotypes that cause SCD have been described, although most are rare [2]. Nine cases of homozygous and compound heterozygote *δβ*-thalassemia have been reported [4, 5].

We report a case of Sicilian (*δβ*)^0^-thalassemia, which is a rare variant of SCD.

## 2. Case Presentation

We report an 18-month-old Caucasian female who presented at 3 weeks of age with an abnormal newborn screen reported as “FS” ( fetal hemoglobin, sickle hemoglobin) indicating the presence of SCD.

Family history included a Southern European ancestry and was negative for SCD/sickle cell trait in family members. There was a remote history of thalassemia trait in maternal grandmother.

Physical examination was unremarkable and laboratory testing by high-performance liquid chromatography (HPLC) reported the absence of Hemoglobin A, predominant presence of Hemoglobin F (89.8%), and the presence of Hemoglobin S (10.2%); this was interpreted as the presence of sickle cell disease. Complete blood count (CBC) revealed a hemoglobin count of 16 g/dl, leukocyte count of 4820 k/uL, and reticulocyte count of 1.5% and MCV was 92.3 fL.

Sickle cell disease counseling was completed and penicillin prophylaxis was initiated.

Repeat HPLC evaluation showed the absence of HbA, HbS 50%, HbA2 2.9%, and HbF at 46.2%. (Table 1).

Globin gene comprehensive analysis was then requested and showed compound heterozygosity for the HbS mutation and a large deletion of the beta-gene cluster that spans the delta-globin* (HBD)* and beta-globin* (HBB)* genes. This large deletion is called the Sicilian form of (*δβ*)^0^-thalassemia; hence this patient has a form of SCD called Sickle-(*δβ*)^0^-thalassemia. Parental testing showed the presence of the sickle cell trait and persistent fetal hemoglobin. CBC results are not available. Patient has been followed at the Sickle Cell Clinic and so far has not experienced any vasoocclusive crises or sickle cell related complications.

The above case portrays the need to accurately diagnose rare hemoglobinopathies which result in SCD, especially when these rare variants are associated with potential symptoms and complications.

## 3. Discussion

*δβ*-thalassemia is characterized by decreased or absent synthesis of the delta- and beta-globin chains with a compensatory increase in expression of fetal gamma-chain synthesis. The condition is found in many ethnic groups but is most common in Greece and Italy. Homozygotes for *δβ*-thalassemia have 100% HbF and, because of the increased synthesis of HbF, may have thalassemia intermedia rather than thalassemia major [6, 7].

The heterozygous form of the condition phenotypically resembles B thalassemia trait but HbA2 is often normal while HbF is elevated varying from 5% to 20% [7].

Since homozygous *δβ*-thalassemia presents an identical HPLC finding as homozygotes of hereditary persistence of fetal hemoglobin of 100% HbF, the clinical findings of mild hemolytic anemia rule in favor of *δβ*-thalassemia rather than HPFH [8]. Family studies also play a role in eliciting the correct diagnosis (thalassemic features).

Sicilian (*δβ*)^0^-thalassemia presents a deletion of 13,379-bp spanning *δ*-IVS2 to a region located 3′ from the *β*-globin gene within an L1 repeat. [9].

Sickle-(*δβ*)^0^-thalassemia is a rare SCD variant that has been sparsely reported worldwide [4, 5]. These cases were described to have mild microcytic anemia, as well as SCD complications which include multiple episodes of VOC (in some cases this occurred prior to diagnosis), osteomyelitis, multifocal avascular necrosis, cholelithiasis, and osteonecrosis [4, 5].

Eliciting diagnosis in suspected rare variants of SCD cases is crucial, as it enables formulation of a comprehensive plan of care.

In our patient, the HPLC demonstrated the absence of HbA, as expected for this genotype. Globin gene comprehensive analysis elicited the diagnosis as reported in Table 2. The HbF level is elevated, even for the patient's young age, but it is expected to decrease between 10 and 20% by adulthood. The HbA2 is low and will remain low throughout life because the patient has only one remaining delta-globin* (HBD)* gene.

Patients with Sickle-(*δβ*)^0^-thalassemia have normal hemoglobin levels and/or mild anemia and normal or slightly increased reticulocyte counts. The blood smear has typical thalassemic features, including hypochromia, target cells, basophilic stippling, occasional microcytes, and red cell fragments [10]. Hemoglobin electrophoresis shows HbS with elevation of HbF.

Sickle-(*δβ*)^0^-thalassemia is different from compound heterozygosity for HbS and gene-deletion hereditary persistence of fetal hemoglobin (HPFH), which is typically asymptomatic.

In reported literature, patients with Sickle-(*δβ*)^0^-thalassemia have had splenomegaly, spontaneous rupture of an enlarged spleen, significant perioperative complications, and mild-moderate vasoocclusive crises; pregnancy reported in two patients was complicated by occurrence of vasoocclusive crises and febrile illness [10–13]

This case shows the complexity that can accompany interpretation of the newborn screening reports for sickle hemoglobinopathy, as well as the need for an accurate diagnosis which is crucial in estimating disease severity and planning appropriate treatment.

Screening newborns for rare genetic diseases was initiated approximately forty years ago [14].

In 2002, the federal health resources and services administration's Maternal and Child health Bureau tasked the American College of Medical Genetics (ACMG) to develop guidelines for newborn screenings; at that time, some states screened for as few as four conditions and others as many as 50 [15, 16]. It is important to note that further testing which includes gene sequencing studies are needed to elicit the presence of rare variants of SCD.

*In conclusion*, our patient has maintained an unremarkable clinical course up till present times; however this may be related to age. Management strategy has involved the National Heart Blood Lung Institute (NHLBI) guidelines for the management of sickle cell disease (though not specified for this rare variant of sickle cell disease)

Penicillin prophylaxis was recommended; routine health examinations are ongoing.

In prior reported cases of Sickle-(*δβ*)^0^-thalassemia, hydroxyurea was utilized in 2 patients who had multiple vasoocclusive crises [5]. Hydroxyurea utilization in this case will depend on disease severity/SCD complications.

## Figures and Tables

**Table 1:** HPLC and CBC results in patient.

	6 days	6 months	12 months
HPLC values			
HbA	0	0	0
HbF	89.8	46.2	41.2
HbA2	0	2.9	3.3
HbS	10.2	50.9	55

CBC values			
Hemoglobin (g/dl)	16	14.1	15.4
MCV (fL)	92.3	67.1	67.5
ARC (×10^3^/ml)	n/a	0.09	0.1

**Table 2:** Globin gene comprehensive analysis.

Genotypes	
HBB genotype	*β* ^S^/(*δβ*)^0^

*Genetic significance*. This patient is compound heterozygous for the missense mutation, c.20A>T, in *HBB*, and a 13.4 kb deletion that includes the *HBB* and *HBD* genes, NG_000007.3:g.64336_77738del13403 (13.4 kb Sicilian (*δβ*)^0^–thal deletion).
